# User experiences with a mobile health app for self-management of diabetes and hypertension in Ghana: a qualitative study

**DOI:** 10.1080/07853890.2025.2517395

**Published:** 2025-06-13

**Authors:** Pearl Aovare, Erik Beune, Amos Laar, Nicolas Moens, Eric. P. Moll van Charante, Charles Agyemang

**Affiliations:** ^a^ Department of Public and Occupational Health, Amsterdam UMC, Amsterdam Public Health Research Institute, University of Amsterdam, Amsterdam, The Netherlands; ^b^ Department of Population, Family and Reproductive Health, School of Public Health, University of Ghana, Legon, Accra, Ghana; ^c^ VU University Athena-Institute, Economics, eHealth, and Digital Transformation, Amsterdam, The Netherlands; ^d^ Department of General Practice, Amsterdam UMC, University of Amsterdam, Amsterdam, The Netherlands; ^e^ Department of Medicine, Johns Hopkins University School of Medicine, Baltimore, MD, USA

**Keywords:** Diabetes, hypertension, mobile health (mHealth), patient engagement, self-management, Digital health, Chronic Disease Management, Ghana

## Abstract

**Background:**

Cardio-metabolic disorders like diabetes and hypertension are increasingly common in low- and middle-income countries, including Ghana, straining healthcare systems. Mobile health (mHealth) applications offer potential for improving remote monitoring, patient engagement, and communication with providers. However, their implementation in Ghana remains limited and complex. This study explored user experiences with an mHealth app for self-managing diabetes and hypertension, and its perceived impact on care quality.

**Methods:**

A qualitative study was conducted with 20 participants from two healthcare facilities in Ghana using an mHealth app to manage diabetes or hypertension. In-depth interviews, guided by the Technology Acceptance Model (TAM), were audio-recorded, transcribed, and analyzed using thematic analysis.

**Results:**

Participants reported that the app improved self-management, care coordination, and communication with providers. Valued features included medication reminders, appointment scheduling, and health monitoring, which fostered empowerment and engagement. The app also promoted healthier lifestyle choices. However, challenges such as data security concerns, mobile phone literacy, poor internet access, and data costs were noted.

**Conclusion:**

mHealth apps can enhance self-management and perceived care quality by supporting patient engagement and provider communication. To maximize their impact, challenges around digital literacy, connectivity, and data security must be addressed. Policymakers should promote secure, equitable, and sustainable integration of mHealth technologies into the healthcare system.

## Introduction

The rate of cardio-metabolic disorders, such as diabetes mellitus and hypertension, is rising in low- and middle-income countries, including those in Africa, such as Ghana [1]. For instance, in 2021 alone, over 306,000 people under 60 years of age died due to diabetes in sub-Saharan Africa, with prevalence rates escalating from 4.5% to 7.2% among adults over a 5-year period [2]. Similarly, hypertension has affected 106 million adults worldwide in 2016 doubling from 46 million adults in 1990 [3]. This growth has been attributed to urbanization, transition in epidemiology, and aging, as diabetes and hypertension remain pervasive health concerns affecting millions worldwide. Global health statistics show that the prevalence of these conditions is putting significant strain on healthcare systems. A 2019 WHO survey reported that Africa had the second lowest diabetes-related expenditure (US$ 13 billion), representing just 1% of global spending [4]. In Africa, diabetes costs cover drugs, diagnosis, and consultations, but only 36% of countries have essential medicines in public hospitals [5]. Managing both diabetes and hypertension requires tailored interventions. mHealth solutions can bridge healthcare gaps by enhancing patient engagement, enabling remote monitoring, and improving care coordination, leading to better health outcomes [6]. In Ghana, diabetes and hypertension frequently co-occur, contributing to a rising burden of non-communicable diseases [7]. A systematic review and meta-analysis estimated that the prevalence of diabetes among adults is approximately 6.5, while hypertension affects about 30.3% of the adult population [8,9]. Although national-level data on co-occurrence are limited, studies indicate a high hypertension prevalence among patients with type 2 diabetes. For instance, a study at Korle–Bu Teaching Hospital reported that 79.9% of individuals with type 2 diabetes also had hypertension [10]. Similarly, research conducted in the Ejisu–Juaben Municipality found that 76.7% of diabetic patients had hypertension as a comorbidity [11]. This dual burden places significant strain on healthcare resources, especially since only a fraction of patients receive adequate care and treatment.

Technological advancements have created opportunities to leverage mobile devices for health purposes. Mobile health (mHealth) represents a shift in healthcare delivery, offering solutions for disease self-management, remote monitoring, and patient engagement. Its integration into chronic disease management enhances accessibility, promotes self-care, and improves patient–provider communication [12].

Recent studies have demonstrated the effectiveness of mHealth interventions in chronic disease management, with systematic reviews highlighting their role in improving patient adherence, glycemic control, and blood pressure regulation. A study by Ni et al. [13] synthesized evidence on mobile phone-based interventions and found that mHealth tools enhance patient engagement and support shared decision-making, particularly in the management of chronic conditions. These interventions empower individuals to take an active role in their care, leading to improved treatment adherence and healthier behaviors [14]. However, while the benefits of mHealth are well-documented, implementation challenges persist, particularly in LMICs like Ghana. Despite the increasing adoption of mHealth solutions in LMICs, there is still a gap in the current understanding how patients with these conditions perceive and interact with mHealth tools, as well as their preferences and challenges regarding these technologies [13–16]. It is important to tailor interventions to the specific needs of individuals with diabetes and hypertension. Personalized and culturally sensitive approaches are vital for the success of mHealth interventions. This underscores the importance of ongoing research to optimize the effectiveness of these technologies in managing chronic conditions.

Recent studies, such as the ComHIP intervention in Ghana, have shown that integrating mHealth tools into community-based care improves hypertension management by enhancing treatment adherence and blood pressure control [17]. Similarly, Laar et al. highlighted mHealth’s role in promoting self-management, while the Text2Heart study demonstrated its potential for continuous patient support [18,19]. Furthermore, while existing studies report improved outcomes, they often lack detailed exploration of user experience and do not assess whether Ghana’s current mHealth infrastructure is equipped to support scalable and equitable implementation. As Mishra et al. note, behavioral and contextual challenges such as patient motivation, digital literacy, and system-level barriers underscore the importance of user-centered approaches. Therefore, there is a need to explore how patients with chronic conditions in Ghana specifically engage with, perceive, and derive value from mHealth tools within real-world healthcare settings.

To better understand patients’ perceptions of mHealth tools for managing diabetes and hypertension, this study applied the Technology Acceptance Model (TAM) as its guiding theoretical framework. Developed by Davis, TAM is widely used to explain user adoption of technology, emphasizing two key constructs: Perceived Usefulness (the extent to which a user believes a technology will enhance their performance) and Perceived Ease of Use (the degree to which a user finds the technology effortless to use). Given the study’s focus on patient engagement with an mHealth app, TAM provides a structured approach to explore how these perceptions shape app usage, satisfaction, and long-term adherence to digital health interventions.

This study addresses these gaps in understanding user perceptions and long-term engagement, related to mobile care app for diabetes and hypertension management, assessing its impact on self-management and perceived quality of care in a resource-limited setting.

## Methods

### Setting and study design

**Figure 1.**
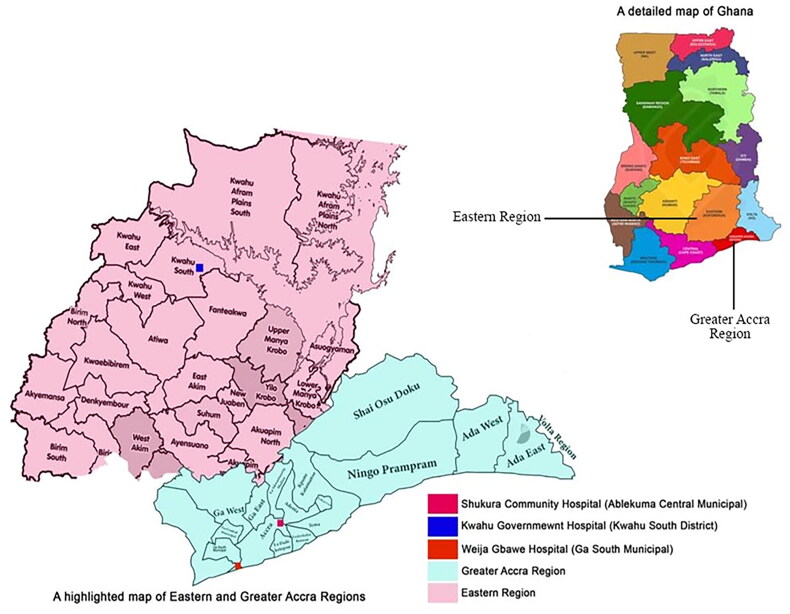
Map of study sites in Eastern and Greater Accra regions, Ghana.

This study involved two healthcare facilities in Ghana’s Greater Accra and Eastern regions (Figure 1) two public facilities specializing in outpatient treatment for type 2 diabetes and hypertension in Greater Accra and Eastern regions were randomly selected for the study. All clinics served people of diverse socio-economic backgrounds. The first clinic, Weija–Gbawe Hospital, is situated in the South-western part of Accra, formerly known as Ga South Municipal Hospital or “Akawe Hospital.” It employs 576 health workers, including 380 clinicians and 196 non-clinicians. Within the Weija–Gbawe Municipality, the hospital sees a daily admission rate of approximately 60 to 100 patients, with over 300 patients attending the outpatient departments (OPDs) and managing around 100–150 diabetes and hypertension patients weekly. The second clinic, Kwahu Government Hospital, is located in the semi-rural Kwahu South district of the Eastern region of Ghana. This district-run academic hospital serves an estimated population of 230,000 people from over 200 communities in the Kwahu municipal and beyond. The hospital offers diabetes care and general healthcare, boasting a substantial diabetes clinic that attends around 200 predominantly type 2 diabetic and 300 hypertensive patients weekly.

These geographically dispersed clinics range from approximately 7.2 km to 151.2 km from each other (Figure 1). We purposefully selected these facilities to ensure diversity in clinics location (urban or rural), patient demographics (age, gender, socio-economic status), facility size and capacity, accessibility and some community characteristics.

Both hospitals have some experience with digital health interventions, including electronic health records and SMS-based patient reminders, but the use of mHealth for chronic disease management remains limited. While Ghana’s healthcare system has explored various mHealth initiatives – particularly in maternal and child health – their integration into chronic disease care is still evolving. Existing mHealth interventions in Ghana, such as SMS reminders for medication adherence and health education programs, have shown promise but are not yet widely adopted across healthcare facilities.

### Overview of the app and its specific functionalities

The app (named: Afya Pro Connected Care app) was part of a pilot implementation. The pilot implementation of the app aimed to evaluate its effectiveness as a mobile health intervention for managing chronic diseases, specifically diabetes and hypertension (https://appadvice.com/game/app/afyapro/1464879369) or https://ecareaccess.org/. Through this pilot, the app’s functionality and usability were assessed to determine its potential for broader implementation in supporting proactive and connected chronic disease management (Appendix 1). The app is a complete healthcare platform that combines tools for patient engagement, care plans, decision support, communication, logistics, finance, and data warehousing. It is enriched with best practices and behavioral change expertise, making it versatile and impactful for diverse healthcare needs, benefiting both healthcare workers and patients. It includes features like real time patient monitoring, a communication platform, medication reminders, health education resources, appointment scheduling, secure data storage, decision support features and user-friendly interface. The app enables healthcare professionals to monitor patients’ health status remotely and facilitates communication between providers and patients utilize the app to track vital signs, receive real-time health data, and engage in timely communication with their patients for effective management of diabetes and hypertension.

Participants received structured education on diabetes and hypertension management delivered by trained nurses including self-monitoring, medication adherence, diet, and physical activity, to complement and support their use of the app’s functionalities, which allowed them to log and monitor these health metrics in real time.

Training sessions were conducted both in-person and virtually, with follow-up support through the app’s built-in messaging system. Users also had access to teleconsultations and periodic check-ins by healthcare providers to address concerns and reinforce adherence.

Despite the benefits of mHealth, access remains uneven due to variations in healthcare infrastructure, internet connectivity, and smartphone use. Digital literacy and language barriers further impact its effectiveness. To truly be effective, mHealth solutions must be adaptable not only in terms of technological compatibility but also in their connectivity requirements, integration with health payment and insurance systems, language and visual design, and levels of complexity suited to users’ digital literacy. Tailoring solutions to these diverse healthcare settings will help ensure equitable access and improved outcomes.

### Study population

Patients from both the Greater Accra and Eastern region of Ghana, aged 18 to 85, were eligible if they used the Afya pro connected care 2.0 app on their phones and had a confirmed diagnosis of type 2 diabetes or hypertension, as documented in their medical records or assessed by healthcare providers. Eligible participants needed to receive outpatient medical treatment for their disease at one of the public clinics for a minimum of one year (Table 1).

**Table 1.** Study participants’ background information.

Patient ID	Sex	Age	Educational status	Marital status	Type of condition	Duration of disease/treatment (years)
1	Male	43	Tertiary	Married	Hypertension	7
2	Female	33	Tertiary	Married	Diabetes	12
3	Male	50	Secondary	Married	Diabetes	20
4	Male	43	Primary	Married	Diabetes	9
5	Female	34	Tertiary	Single	Diabetes	4
6	Male	62	Secondary	Married	Hypertension	15
7	Male	47	Secondary	Married	Diabetes	8
8	Male	52	Tertiary	Married	Hypertension/diabetes	20
9	Female	36	Tertiary	Married	Diabetes	10
10	Male	53	Primary	Married	Diabetes	23
11	Male	36	No education	Married	Diabetes	30
12	Female	49	Secondary	Married	Diabetes	7
13	Female	53	Tertiary	Married	Hypertension/diabetes	23
14	Male	65	Tertiary	Married	Hypertension/diabetes	15
15	Female	57	Secondary	Separated	Hypertension/diabetes	5
16	Male	71	No education	Widower	Hypertension	35
17	Male	68	Primary	Separated	Hypertension	19
18	Female	52	Secondary	Married	Diabetes	12
19	Male	47	Primary	Single	Hypertension	8
20	Female	59	Primary	Married	Diabetes	17

### Recruitment and participation

Initially, we recruited 40 participants through purposive sampling from various healthcare facilities to ensure a diverse sample. We employed a purposive sampling strategy, specifically maximum variation sampling, to capture a wide range of experiences and perspectives on app usage. Key factors included age, socioeconomic status, chronic disease management experience, and cultural attitudes toward technology. Some participants dropped out of the study due to technical difficulties, such as issues with smartphone functionality, including limited storage capacity, slow response times when using the app, battery life problems, and lack of internet access (*n* = 7). Personal circumstances, such as relocation and changes in healthcare facilities (*n* = 9), also contributed to the dropout rate. Additionally, health complications, such as severe symptoms related to diabetes or hypertension that necessitated urgent medical attention, led some participants to prioritize their immediate healthcare needs over continued participation in the study (*n* = 4). Examples include episodes of hyperglycemia or hypertension crises, which required hospitalization or adjustment of medication regimens.

### Participant engagement with mobile app use

Participants were encouraged to use smartphones provided with the AfyaPro Connected Care app, which was downloaded from platforms like the Google Play Store onto their devices by healthcare workers. Healthcare providers facilitated training sessions to help participants navigate the app, understand its features, and learn how to input essential health information, like blood glucose and blood pressure readings. Healthcare providers facilitated two training sessions for each participant, each lasting approximately 45 min. These sessions aimed to familiarize participants with the AfyaPro Connected Care app, covering navigation, essential features, and health data entry methods. The training employed a hands-on approach, using demonstrations and practice exercises to enhance comprehension and retention. In addition, simple, illustrated user guides were provided for reference at home. These educational strategies ensured that participants felt confident in independently using the app, recording health metrics, and accessing app-based resources to support their diabetes and hypertension management. This training empowered participants to use the app independently at home, fostering a sense of control over their health management.

Participants were instructed to use the app at home to record daily health metrics, receive medication reminders, and access educational resources that supported disease management. The app’s real-time connectivity enabled healthcare providers to remotely monitor these inputs, identifying any alarming trends that might require follow-up. When necessary, healthcare workers reached out to participants to provide guidance, address concerns, or schedule appointments for in-person assessments. Community health volunteers further supported this process by assisting participants, ensuring app adherence, and helping them interpret feedback, reinforcing their engagement with the app and its health management capabilities.

Follow-up sessions were conducted bi-weekly throughout the pilot study, allowing healthcare providers to regularly assess participants’ engagement with the AfyaPro Connected Care app. During these sessions, providers reviewed health data inputted by participants, provided personalized feedback, and addressed any challenges, ensuring continuous support and encouragement for effective health management.

### Data collection

In-depth interviews (IDIs) were conducted from August 2022 to January 2023, using a semi structured format using a topic guide to gain a comprehensive understanding of the factors influencing patients’ acceptance and utilization of the Afya Pro Connected Care app in the management of diabetes and hypertension (Table 2) (Appendix 2). The interviews focused on the app’s usefulness, ease of use, functionalities, navigation, and overall usability in managing chronic conditions. Field notes were also taken during the interviews to capture participants’ experiences with the mobile app. These notes included observations, quotes, and context details, helping verify the data, enrich the analysis, and support the research findings as well. IDIs were conducted in English or the local dialect, ‘Twi’, through face-to-face conversations within healthcare facilities to ensure a comfortable setting. Twi interviews were professionally translated and transcribed for accuracy. A bilingual researcher handled the initial translation, with an independent review and back-translation of key sections to ensure consistency to the original responses. Interview durations ranged from 20 to 45 min.

**Table 2.** Interview topic guide.

Topic	Subtopic
Researcher introduction	Introduction interviewer, research group and sign informed consent
Participants introduction	Demographics of health workers
Perceived usefulness and efficacy of mHealth intervention	Value of the mHealth app in managing diabetes and hypertension. Interaction with healthcare providers. Convenience of using the app for communication. Confidence in sharing information with providers through the app. App training and education received.
mHealth services and service satisfaction	App effectiveness: entering data, reminders, viewing information, and scheduling. Impact on diabetes/hypertension management. Blood sugar/blood pressure control. Empowerment and control over health. Satisfaction with remote monitoring and challenges encountered.
Implementation of mHealth app	Features and functions used. Comfort using the app and time commitment. Availability of specialized services through the app. Impact on in-hospital treatment and general care for diabetes/hypertension. Reflections on challenges and recommendations for improvement.

Data saturation, indicating the point at which new information stops emerging, served as the criterion for determining the sample size adequacy [12] was achieved after conducting 19 interviews. An additional interview was carried out to confirm this, ensuring no new themes or insights emerged.

For this study, we focused on issue-specific saturation related to the experiences and challenges faced by participants using the Afya Pro Connected Care app. specifically; we examined themes around app usability, health management behaviours, and the impact of social factors on technology adoption. We aimed to capture a comprehensive understanding of these issues, and the 20 participants provided sufficient richness and diversity in their experiences to meet this goal.

A team of qualitative researchers and data analyst collaborated to review ongoing interview responses, analysing them for repetition or redundancy in themes to ensure a comprehensive understanding of the data. The collaborative nature of this process allowed for a comprehensive evaluation of the data; once the team agreed that no new themes were emerging, saturation was deemed achieved.

### Data analysis

The data analysis utilized Braun and Clarke’s six-phase thematic analysis approach, which included familiarization with the data, generating initial codes, searching for themes, reviewing themes, defining and naming themes, and producing the final report [16]. We employed both deductive and inductive coding methods. The deductive coding followed Davis’s Technology Acceptance Model (TAM) to identify themes related to its constructs, while the inductive approach captured unforeseen subthemes from participant narratives (Appendix 3). This dual strategy enriched our analysis, allowing us to integrate diverse perspectives and insights throughout the process.

Themes in this analysis are defined as overarching patterns or broad concepts derived from the data, which encapsulate significant aspects of participants’ experiences and viewpoints. Subthemes, on the other hand, represent specific clusters of codes identified during the coding process, highlighting particular elements of the data that contribute to the development of these broader themes [20].

Two Researchers engaged in iterative discussions to thoroughly integrate inductive findings into the analysis. Collaboratively resolving coding discrepancies ensured consistent interpretation. Thematic categories summarizing factors influencing mHealth intervention use in diabetes and hypertension management were generated and reviewed. Atlas Ti software version 9 facilitated organized and systematic coding and analysis. We meticulously refined these themes to accurately represent the data and finally, we created a clear and concise report, adhering to SRQR guidelines, and supported our findings with illustrative examples. In conducting and reporting our qualitative research, we adhered to the Consolidated Criteria for Reporting Qualitative Research (COREQ): a 32-item checklist for interviews and focus groups as outlined by Tong et al. [21]. To provide transparent reporting and clear and thorough insights into our research processes and outcomes (Appendix 4).

### Ethical considerations

This study was approved by the Ghana Health Service Ethical Review Committee (GHS-ERC004/08/20). Informed consent for participation was obtained from all participants aged 18 and above. The study did not include minors.

Written informed consent was provided by all adult participants after they received detailed information about the study’s purpose, procedures, and any potential risks or benefits. Participants were informed that participation was voluntary and that they could withdraw at any time without consequence.

This study was conducted in accordance with the ethical principles outlined in the Declaration of Helsinki.

### Consent for publication

Written consent to publish identifying details has been obtained from all individuals involved in the study. All participants were informed about the publication of their data and provided written informed consent specifically for this purpose. Despite this consent, all efforts have been made to anonymize participants to protect their privacy.

## Results

Twenty participants were interviewed, comprising 11 males and 9 females, aged between 33 and 71 years. Educational backgrounds varied: 2 had no education, 5 had primary education, 6 had secondary education, and 7 had tertiary education. Most participants were married [16], with others being single [2], separated [2], or widowed [1]. Regarding health conditions, 10 participants had diabetes, 5 had hypertension, and 5 had both diabetes and hypertension. The duration of their conditions ranged from 4 to 35 years (see Table 1 for further details of the study participants’ background information).

The analysis identified three key themes aligned with the TAM constructs; Perceived Usefulness, Perceived Ease of Use, and External Factors Affecting Acceptance, along with 15 subthemes. Some of these themes were already recognized within the TAM framework during the deductive phase, while others emerged as additional factors influencing mHealth adoption (Table 3).

**Table 3.** Themes and subthemes aligned with the technology acceptance model (TAM).

Themes	Subthemes
1. Perceived usefulness (PU)	**- Perceived benefits of mHealth tools in diabetes-hypertension management** **- Health/behavior outcomes** **- Stress management support** **- Satisfaction with remote monitoring features** **- Influence on patient–provider interactions** **- Perceived benefits of continuous monitoring** **- Trust in virtual consultations**
2. Perceived ease of use (PEOU)	- **Acceptability of the app** **- Feeling in control of health information** **- Increased confidence in health management** **- Family support for app usage**
3. External factors affecting acceptance Challenges and barriers	**- Trust in data security measures** **- Digital literacy and age-related challenges** **- Geographical accessibility** **- Data plans**

### Perceived usefulness

The degree to which individuals believe that using the mHealth app enhances their health management.

#### Benefits of mHealth (perceived benefits of mHealth tools in diabetes hypertension)

Most patients highlighted the advantages and positive outcomes of using mobile health (mHealth) tools, noting their contribution to effective healthcare delivery.

Majority of the participants (*n* = 17) found the mobile health app beneficial for managing their conditions. They appreciated features like medication reminders and appointment scheduling, as well as the ability to input health data for real-time monitoring by healthcare providers.

*The app’s medication reminders and appointment scheduling were very helpful. My family assisted me with using the app, which made managing my health easier. Inputting health data into the app also allowed providers to give me timely feedback, making the app highly beneficial.* ***Participant 3, male 50 years.***

*Yeah yeah, I am able to put my vitals my sugar and blood pressure readings in the app, I also know when I should go to see my doctor through the app, it has also reduced cost for me, because I don’t travel unnecessarily to the clinic again* ***. Participant 7, male, 47 years***

#### Health/behavior outcomes

Participants noted that the app offered valuable guidance on dietary habits, helping them manage their health more effectively. Interviews also revealed that users connected the app to improvements in their diet, weight loss, and overall well-being.

*The app provided helpful information on staying fit through exercise and maintaining a healthy diet, emphasizing less carbohydrate intake, more vegetables, and smaller portion sizes for carbohydrates* ***. Participant12, female 49 years.***

*Yes, I have been able to reduce my weight through constant checks, information on how to plan a better diet has really contributed to my weight reduction, and today I am so happy.*

***Participant 7, male, 47 years***

#### Stress management support

Participants noted that the app eliminated the need to travel for healthcare, which was especially beneficial in areas with limited access due to distance or other obstacles. It simplified the process of managing their healthcare needs, allowing for convenient oversight of various aspects of their care.

*Using the app has been incredibly beneficial. It helps me stick to my medication plan, eat healthily, exercise regularly, and prioritize sleep, all of which have reduced my stress.* ***Participant 2, female, 33 years.***

### Patient experiences with remote health monitoring

Most participants had positive experiences with remote health monitoring, appreciating the ability to share data with healthcare providers. They valued the continuous monitoring and follow-up, which enabled proactive interventions and timely treatment adjustments, leading to better health outcomes and increased satisfaction.

#### Satisfaction with remote monitoring features

Participants reported high satisfaction with remote monitoring, which boosted their confidence in self-management skills, enhanced their engagement with healthcare workers, and motivated active participation. This may suggest a positive impact on healthcare service utilization and potentially may improve their overall quality of life.

*It has really done a good job on that, I talk to my providers every day, and am really updated on progress with my health; I remember how they follow up on me to take my medications almost on weekly basis, this has really kept me on check.* ***Participants.13, female, 53 years***

*I now enjoy most of the things we do, especially on the app without having to travel a distance, and communication with the nurse have also improved, they check on me, check on drug intake, advise me to eat well and exercise to keep fit.* ***Participant.15, Female 57 years.***

### Influence of mHealth on patient–provider interactions

According to patients, the mobile app improved their interactions with the care provider, fostering better communication and engagement. Patients claimed they actively participated in discussions, leading to more informed decision-making and enhancing the overall patient–provider relationship.

#### Changes in communication dynamics

The participants stated the app has changed how patients and healthcare providers interact, making it more collaborative. Now, patients could share their treatment preferences and talk openly with their providers. According to patients this can improve communication, may help to focus on health goals, and to put patients at the center of care.

*Thanks to the app, it is now much easier to work with our healthcare providers. Communication has improved, I am more focused on my health goals, and the care I receive has gotten better.* ***Participant 3, male, 50 years***

*The app has made healthcare more accessible for me, my doctor now reaches out regularly to check on my health and treatment. This has been incredibly beneficial to me, and I’m eager to keep using it.* ***Participant. 7, male, 47 years***

#### Perceived benefits of continuous monitoring

Furthermore, Patients reported that continuous monitoring improved their health literacy, strengthened communication with healthcare providers, enhanced self-management skills, facilitated personalized care, and promoted long-term health management.

*Yes the app so far is the best, it has done well, imagine you wake up and you are constantly reminded to take your medications and even you get doctor can talk to you on the app and also remind you to book your appointments on the app, is a very good source of reminder for me as long as my health is always a concern.* ***Participant 9, female 36 years***

*It has really done a good job on that, I talk to my providers every day, and am really updated on progress with my health; I remember how they follow up on me to take my medications almost on weekly basis, this has really kept me on check* ***. Participant 13, female, 53 year***

#### Trust in virtual consultations

Some participants found virtual consultations helpful because they trusted the process, appreciating the ease of accessing specialized services, maintaining ongoing care, saving on travel costs, and receiving more focused attention from healthcare providers.

*I trust the nurse’s guidance and follow their instructions without needing to see them in person.*

*This has really improved my health and kept me on track.* ***Participant 10, male 53 yewars***

*I trust the app to stay in contact with my providers, the app has the ability to track my results, remind me of appointments, and offer reliable online consultations when I’m unwell.*

***Participant 12, female 49 years.***

2. Perceived ease of use – The extent to which individuals believe that using the mHealth app is free of effort.

#### Acceptability

Clear guidance on health tasks was reported, likely contributing to the perception of the app as user-friendly and intuitive. Additionally, the app played a key role in enhancing users’ understanding of their medical conditions by providing accessible information and resources. This empowerment encouraged individuals to take a more active role in managing their health. *Much confidence, I can easily use it without any help, I read and use the instructions in it, to share my information such as my weekly results of BP and sugar reading****. Participant11, male, 63 years***

*I can utilize all the functions, entering information, responding to reminders, viewing data, and scheduling appointments with the doctor. My relatives also use all these features with ease* **.**

***Participant 8, male, 52 years.***

### Patient’s sense of empowerment in self-managing health through mHealth

Participants felt empowered in managing their health through the mHealth app, which provided easy access to information, regular monitoring, and opportunities for active engagement. This increased their confidence and motivation to maintain their well-being.

#### Feeling in control

Most participants (*n* = 18) emphasized how the app’s comprehensive resources enhanced their confidence and ability to make informed decisions, allowing them to proactively manage their health choices and behaviors.

*Yes, I am very empowered now, because I know about some of the complications with my*

*Illness now, I can also interpret my readings now.* ***Participant 11, male 63 years***

*I strictly follow the app’s guidance with the help of my providers. The information alerts on diet, salt reduction, medication, and exercise were very useful for managing my condition.* ***Participant 1, male, 43 years***

#### Increased confidence in health management

Many participants experienced increased confidence in managing their health conditions through their engagement with the app. This was driven by heightened motivation and active participation. The ability to monitor sugar levels empowered them to take a more informed and proactive approach to their health. The app’s real-time feedback allowed timely adjustments to behaviors and treatment plans, fostering a sense of accountability and ownership over their health outcomes.

*I am able to self-management my condition now with the help of the app. Such as how to check my own blood pressure and sugar levels insulin, interpret the results and also make good decisions about my health, at first I will wait till the nurse checks and then tell me what it is*.

**Participant16, male 70 years.**

*Very convenient, because now we get to communicate even more often than before, and they get more involved with my health. Before the app, I see we used to be reluctant but now we are so curious to know more about what is happening to us.* ***Participant 20, female, 57 years.***

#### Family support

Several participants emphasized the role of family support in using the app, highlighting assistance with medication reminders, appointment scheduling, and data entry. This support eased their burden, fostered a sense of collective responsibility for health management, and underscored the importance of strong support networks for effective healthcare delivery.

*My family checks my blood sugar early, helps with my insulin injections, and reminds me about setting up appointments through the app. This support helps me stay on track and makes it easier to manage my health.* ***Participant 3 male, 50 years***

*I am okay using the app, and my husband has been helping me with it a lot, he also comes to this facility, so he helps me use it very well at home, especially areas that am not able to use the app effectively, example is sometimes booking my appointments for consultation can be difficult.* ***Participant 10 male, 53 years***

## External factors affecting acceptance

### Challenges and barriers

#### Concerns about data security measures

While not all participants voiced concerns, several (*n* = 5) expressed apprehension about the security of their personal health data in mHealth app, fearing unauthorized access or third-party misuse. They were particularly worried about potential data breaches, lack of transparency, and identity theft. For these individuals, data security was an important issue that influenced their overall trust in the app. However, reassurances from healthcare providers about confidentiality, the implementation of robust security measures, and user control over data access helped alleviate most of these concerns.

*Yes, sometimes we are scared that because the app is an online thing your data might be leaked out in the public, but they have assured us and we hope nothing goes wrong.* ***Participant 16, male 71 years.***

*Initially I was afraid that sharing my information through the app might be shared with a third party, but with time after using the app, am much confident that only providers sees the information and will use it for its intended purpose.* ***Participant 12, female, 49 years***

#### Digital literacy and age-related differences in mHealth use

Participants aged 36–50 years showed varying levels of digital literacy when using the mHealth app. Some experienced difficulties with navigating complex interfaces, understanding technical terms, and handling the devices, particularly among older or less tech-savvy individuals within this group. Many relied on family members or healthcare providers for assistance, limiting their ability to manage their health independently. However, over time, some participants (*n* = 8) reported noticeable improvements in their ability to use the app. This learning effect was facilitated by user-friendly features introduced over time, such as simplified navigation, in-app tutorials, and support from healthcare staff. Additionally, in-person workshops, help desks, and instructional materials provided further guidance, enabling participants to gradually gain confidence and independence.

*Using the app is really challenging for those of us without an educational background. Without my daughter’s help, I could not use it. Maybe they should consider a version that speaks the local language to assist with entries and tasks independently.* ***Participant 9, female, 36 years***

*Yes very, you know I am not so good with this phone use so my kids help me through the process and it has been easy for them, I am confident too in the app* ***. Participant 3, male, 50 years.***

#### Geographical accessibility and poor internet connectivity in remote areas

Participants reported that internet connectivity in remote areas hinder the use of mHealth apps. Limited healthcare infrastructure, unreliable networks, and high data costs disrupt the app usage, leading to interrupted health monitoring and reduced user engagement.

*With the challenges, like I mentioned earlier, some of us stay in areas where the internet Connectivity can go off and not come at all or very slow at times and could affect us using the app to communicate.* ***Participant 1, male 43 years***

*Yes because it is an app that is connected to the internet, occasionally we always have network issues interrupting the app and its daily usage.* ***Participant 15, female, 57 years***

#### Costly data plans

Participants identified the cost of mobile data as a barrier to regular use of mHealth apps, especially in areas with limited internet connectivity. They further suggested creating an offline version of the mobile app for cost-effectiveness and convenient to use.

*Data is becoming expensive to buy and these app are always online using data, so maybe we need another solution, which does not have to be connected to the internet before it works.*

***Participant 3, male 50 years.***

*I really like what the app is doing now, the only thing I might want is an app that does not use data, which make it less expensive, because you have to buy data to be able to be online to use the app.* ***Participant 6, male 62 years.***

## Discussion

Our qualitative study in Ghana explored the experiences of patients with diabetes and hypertension using an mHealth app, and their perspectives on its impact on quality of care. The findings highlight that the mHealth app empowered participants to manage their health, particularly through valued features like medication reminders, appointment scheduling, and health monitoring. These functionalities not only enhanced self-management of diabetes and hypertension but also improved care coordination, engagement, and interactions with healthcare providers. However, participants also mentioned challenges regarding data security and mobile phone literacy, which may impede the app’s full potential in improving patient outcomes and healthcare delivery.

These results align with a study by Arshed et al. [22], which similarly reported enhanced patient engagement, medication adherence, and self-care activities through mHealth interventions. Additionally, our findings correspond with the constructs of the TAM, particularly Perceived Usefulness and Perceived Ease of Use. Participants found the app useful for improving chronic disease self-management, facilitating remote monitoring, and enabling better patient–provider interactions, demonstrating strong Perceived Usefulness. The app’s user-centered design and intuitive interface also contributed to Perceived Ease of Use, as many participants reported minimal difficulty in navigating its features, encouraging continued use and acceptance. Moreover, participants’ ease of adopting the app’s functionalities also shows the importance of user-centered design in promoting technology acceptance and utilization. The app’s features, such as support for dietary modifications and stress reduction, were generally well-received, suggesting potential benefits for healthier lifestyle choices and improved well-being. This aligns with findings from Zhang et al. [23], who reported that users found their diabetes management app intuitive and helpful for tracking blood sugar levels, medication use, and diet. Overall, the user-centered design of the app contributed to its acceptance and effective use, indicating potential for improving diabetes control and lifestyle modification among users.

Theories such as the Empowerment Theory and Social Cognitive Theory further provide a framework for interpreting our findings [24]. The Empowerment Theory suggests that individuals gain control over their health through increased knowledge and skills [25,26]. Our findings –emphasizing user empowerment, active participation, and enhanced patient–provider interaction- are consistent with this theory. Similarly, Social Cognitive Theory supports these findings by highlighting the importance of self-efficacy, observational learning, and reinforcement in behavior change. The app’s continuous feedback and remote monitoring features may have fostered users’ self-efficacy In addition, participants’ expressed willingness to continue using the app reflects the TAM construct of Behavioral Intention to Use.

From the patient’s perspective, the app played a key role in enhancing the quality of care by fostering active patient participation and improving patient–provider interactions. Participants reported feeling empowered through increased access to information and greater involvement in their healthcare journey. Remote monitoring and virtual consultations were particularly valued, facilitating continuous contact with providers and better-informed decision-making. These findings corroborate studies by Kapeller et al. [27], and Vudathaneni et al. [20], which emphasize the role of mHealth in in improving healthcare experiences and patient outcomes. The positive attitudes expressed by participants toward the app align with the TAM construct of Attitude toward Using.

However, despite these benefits, several barriers to widespread adoption were identified, including concerns about data security, digital literacy gaps, geographical accessibility, and costs associated with mobile data. These challenges support previous findings by Zakerabasali et al. [28]. Addressing these barriers is crucial to ensuring equitable access to mHealth technologies. These challenges relate directly to the TAM concept of External Variables, which influence users’ perceptions of usefulness and ease of use, ultimately affecting technology acceptance.

When comparing our findings to previous studies, several differences and similarities emerge. Our study highlighted the value of practical features like medication reminders and appointment scheduling for self-management and care coordination. Other studies, such as Lee et al. [29], focused more on remote monitoring, reflecting different aspects of mHealth benefits. While our participants reported feeling empowered, Addotey-Delove et al. [30] found that healthcare providers primarily viewed mHealth as a monitoring tool rather than an empowerment tool for patients.

Our study has some limitations. Despite a high dropout rate (50%) from the initial list of participants recruited for the interviews, we conducted a detailed analysis of the remaining 20 participants from the two intervention sites. Although selection bias cannot be ruled out those who remained engaged may differ in digital literacy or motivation – the retained participants were diverse in age, socioeconomic status, and chronic disease experience, supporting some generalizability within our qualitative framework. Additionally, our study primarily reflect short-term user experiences, limiting our ability to assess long-term engagement and sustained behavioral changes.

A key strength of our study was the combination of thematic analysis with a theory-driven framework the TAM, which enabled us to contextualize patient experiences within an established model of technology adoption. Additionally, the in-depth exploration of user experiences with the Afya Pro Connected Care app provided valuable findings in usability, user satisfaction, and perceived benefits.

## Conclusion

Our study demonstrates that the Afya Pro Connected Care app improved user engagement and health management among patients with diabetes and hypertension in a resource-limited setting. Key benefits included enhanced empowerment, better access to information, real-time health monitoring and feedback, and strengthened support networks.

The study makes a novel contribution by providing empirical evidence on the role of mHealth in enhancing patient engagement and self-management, particularly in LMIC settings. By linking our findings to the TAM, we offer new insights into factors influencing mHealth adoption, including perceived usefulness, ease of use, and behavioral intention.

Despite these benefits, challenges such as data security, digital literacy, and access barriers must be addressed. We recommend that policymakers and healthcare providers implement clear data protection policies/protocols, invest in digital literacy programs, and support the design of mHealth interventions that are inclusive and user-centered. These measures are critical to maximizing the potential of mHealth technologies to promote equitable and effective healthcare delivery.

## Supplementary Material

Appendix one.docx

Appendix Four.docx

Appendix_Three_Editable.docx

Appendix Two.docx

## Data Availability

The data supporting the findings of this study are qualitative in nature and consist of transcribed and coded interview data. Due to ethical and privacy concerns, raw data cannot be made publicly available. However, quotations from the transcriptions have been used in the results section of the article to support the findings. For access to further data or details regarding the study, please contact the corresponding author. This study follows the Taylor & Francis Share upon Reasonable Request policy.
