# Supplementary material for: User experiences with a mobile health app for self-management of diabetes and hypertension in Ghana: a qualitative study
Source: Ann Med. 2025 Jun 13;57(1):2517395. doi: 10.1080/07853890.2025.2517395 (PMC12168410; doi:10.1080/07853890.2025.2517395)

**Appendix 1 Background information about the mobile Health application (AfyaPro connected care)**

AfyaPro Connected Care is a versatile healthcare management platform, offering patient engagement tools, clinical care plans, decision support, and more. Its enriched content and incorporation of telemedicine represent significant upgrades, empowering both patients and healthcare providers for proactive remote care.

<https://web.facebook.com/AfyaPro2.0/?_rdc=1&_rdr>

The mobile app streamlines healthcare management for medical teams by providing efficient access to patient data. It facilitates real-time monitoring, seamless communication, appointment booking, and alerts for abnormal values. Additionally, it supports multiple sensor integration, insulin dosage calculation, and patient education through shared materials, enhancing overall readability and user experience. <https://appadvice.com/app/afyapro/1464879369#google_vignette>


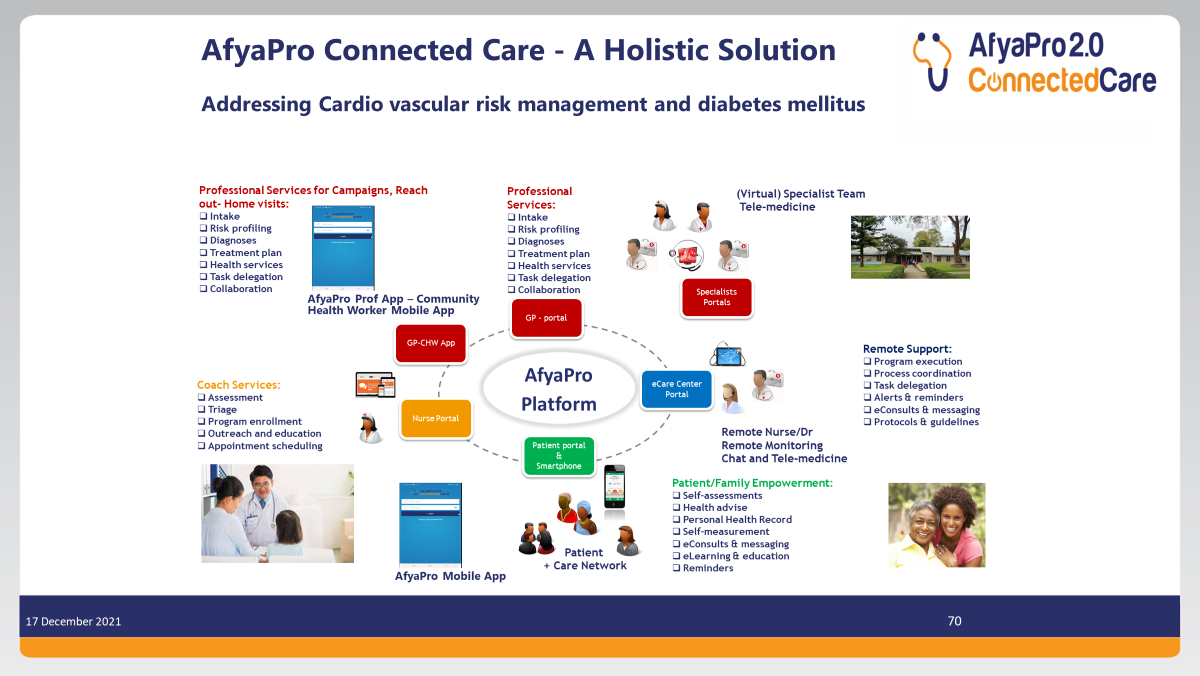


Summary of solution principles

**The Mobile Health APP and key functionalities**


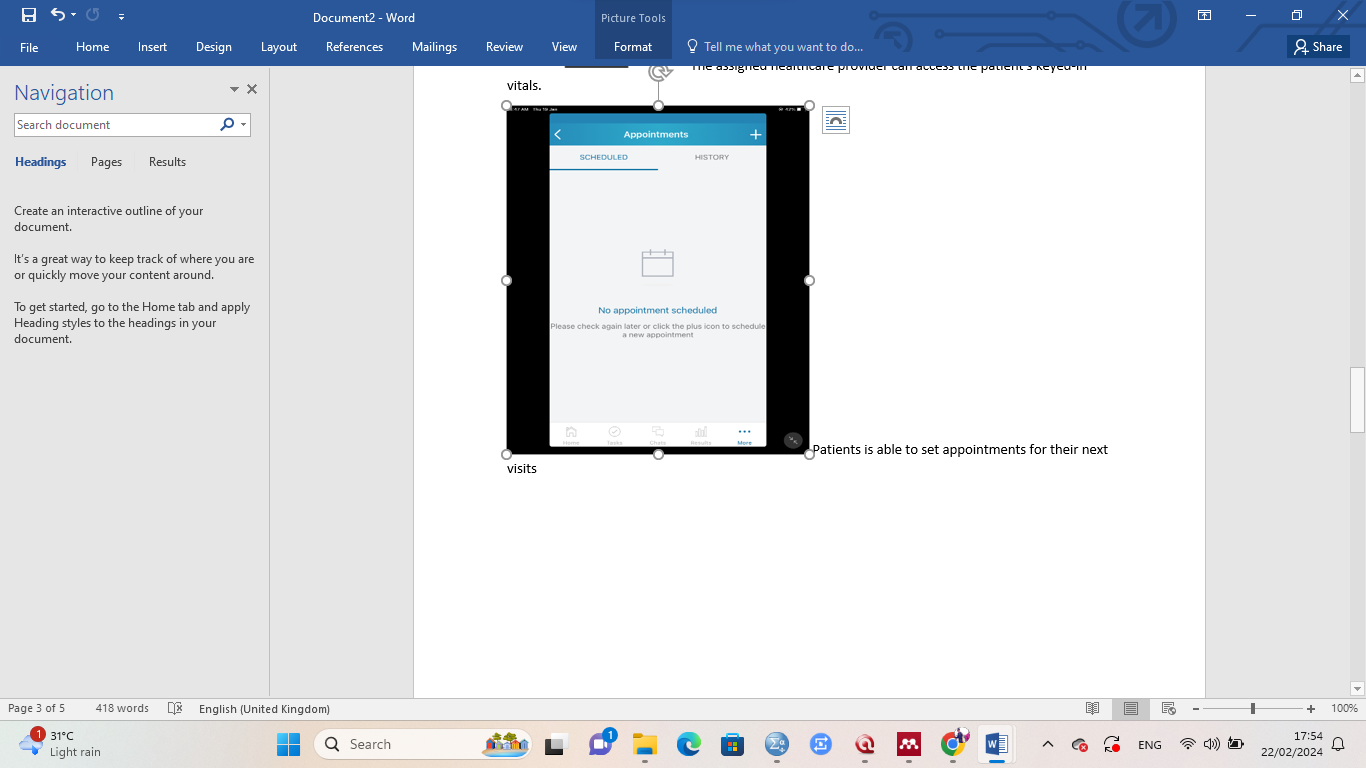

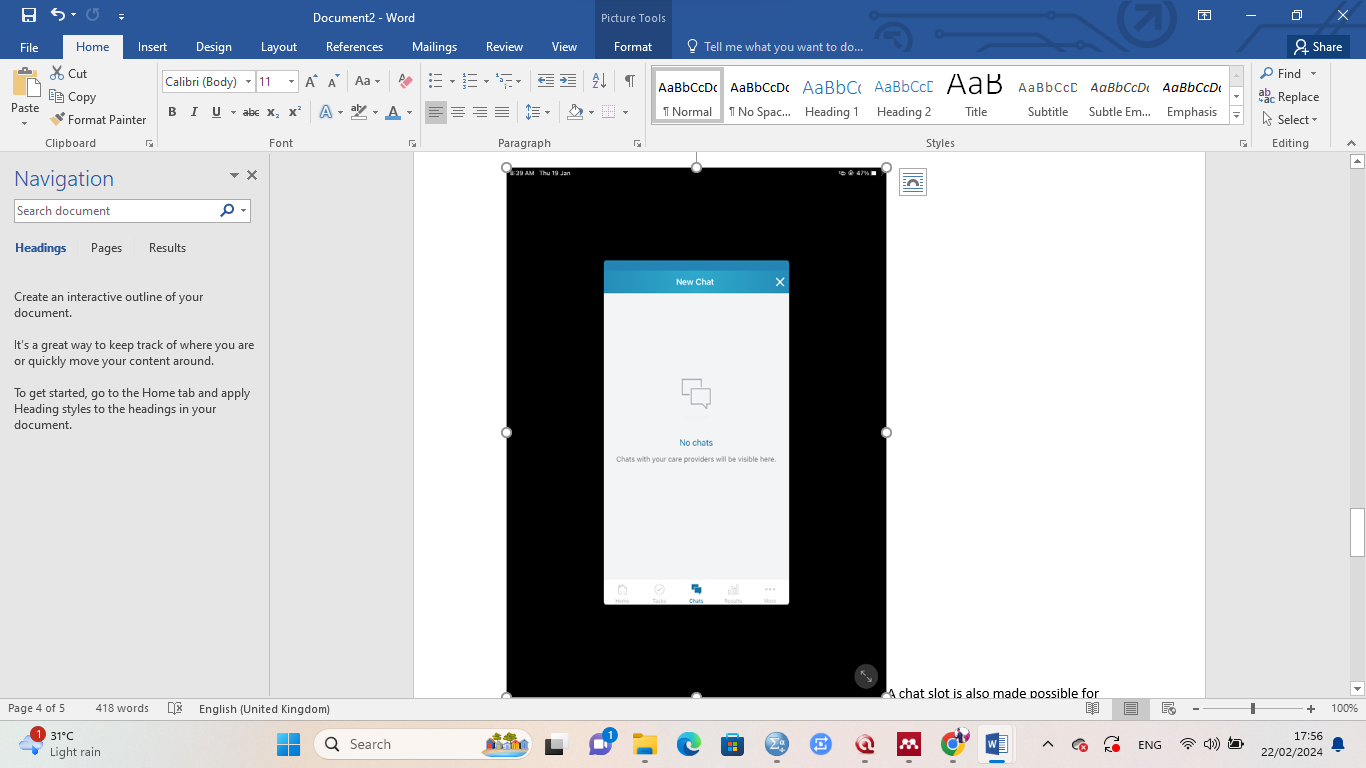

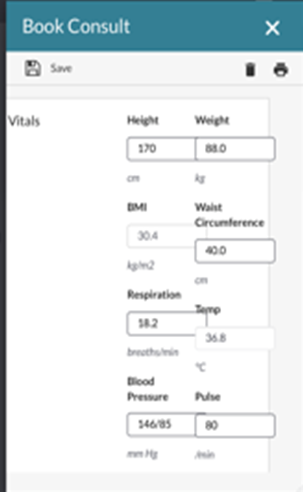


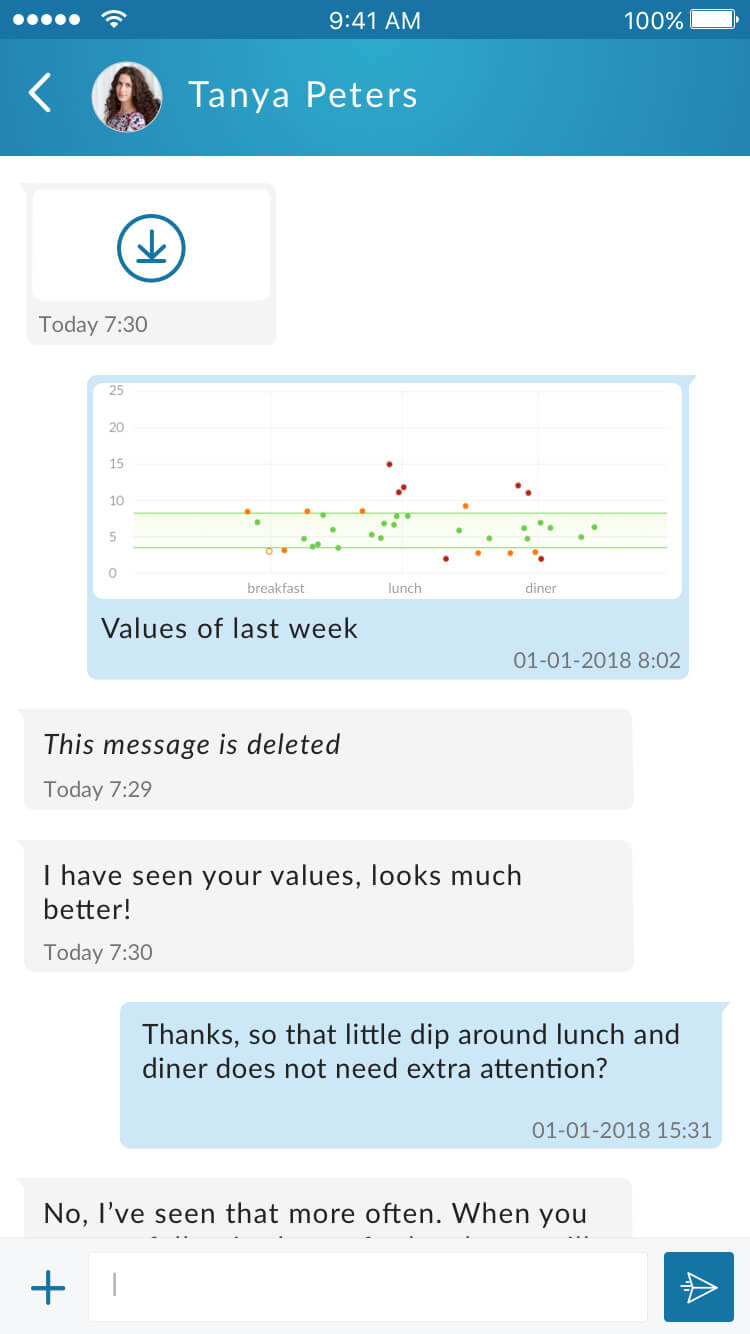


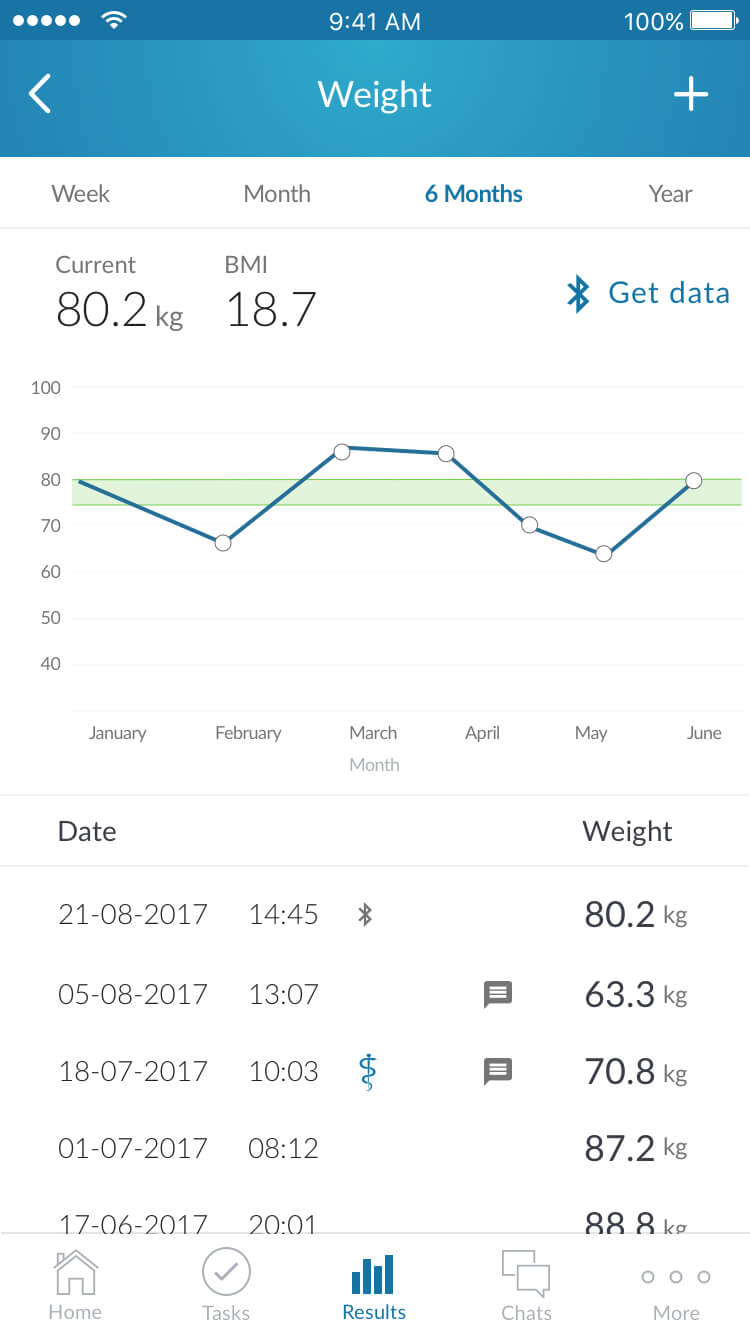

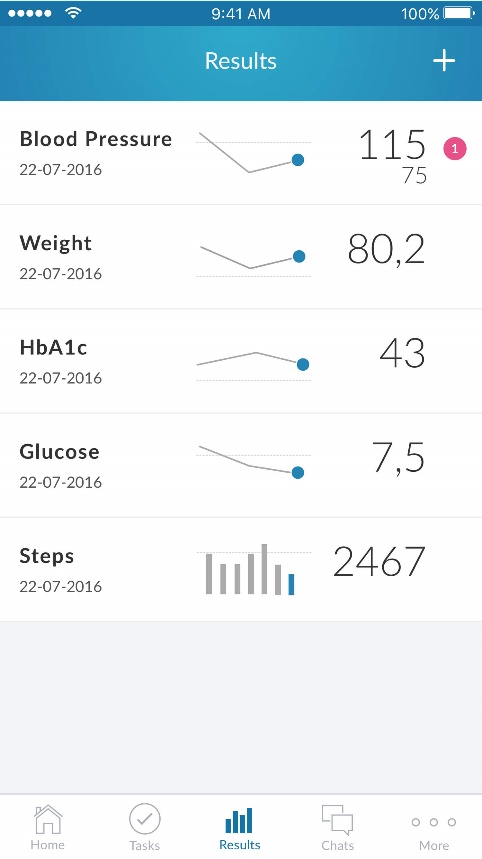

Supplement: Appendix one.docx [file IANN_A_2517395_SM8796.docx]
