# Supplementary material for: User experiences with a mobile health app for self-management of diabetes and hypertension in Ghana: a qualitative study
Source: Ann Med. 2025 Jun 13;57(1):2517395. doi: 10.1080/07853890.2025.2517395 (PMC12168410; doi:10.1080/07853890.2025.2517395)
Supplement: Appendix_Three_Editable.docx [file IANN_A_2517395_SM8794.docx]

**Perceived Usefulness**

**External Variables**

**Actual System Use**

**Behaviour intention to Use**

**Attitude towards Using**

| **Perceived Ease of Use** |
| --- |
|  |

**Appendix 3 (Technology acceptance model. (Davis, 1989)**
