# Supplementary material for: User experiences with a mobile health app for self-management of diabetes and hypertension in Ghana: a qualitative study
Source: Ann Med. 2025 Jun 13;57(1):2517395. doi: 10.1080/07853890.2025.2517395 (PMC12168410; doi:10.1080/07853890.2025.2517395)
Supplement: Appendix Two.docx [file IANN_A_2517395_SM8793.docx]

***Appendix 2; Interviewer guide to assess the management of diabetes and hypertension patients***

***Introduction***

My name is Pearl Aovare, I am from the University of Amsterdam. I am exploring Patient Perspectives: An In-Depth Examination of mHealth Impact on Diabetes-Hypertension Management. We aim to gain valuable insights that can contribute to the enhancement of care quality for patients dealing with diabetes and hypertension, as well as to support healthcare workers in their work to also improve diabetes and hypertension management. We are here to learn from your experiences with using mobile health technologies for managing diabetes and hypertension. we aim to understand how these technologies have influenced your healthcare journey. Your valuable insights will guide us in improving support systems for better overall healthcare. We would like to conduct the interview in approximately 45 minutes and would be happy if we can audio record it. I hope this is possible. Your responses will remain confidential, and the information will not be linked to specific practice sites or individuals. Your participation in this study is voluntary. You may request at any time that we stop the interview or turn off the audio recording. Do you agree?

| Date of interview: | 1. DD | 1. MM | 1. YYYY |
| --- | --- | --- | --- |

| Respondent ID: | Phone No: | *(Intervention facilities) 1/2* |
| --- | --- | --- |

1. Facility: [1] Kwahu Hospital [2] weija-Gbawe Hospital

1.**RESPONDENT’S BACKGROUND**

| 1. Age (in years): |
| --- |
| 1. Sex: [1] Female [2] Male |
| 1. Marital status: [0] Never married [2] Married [4] Widowed    - - 1. [1] Cohabiting [3] Divorced/Separated |
| 1. Religion: [0] None [1] Christian [2] Muslim [3] Traditional African    - 1. Other: |
| 1. Highest level of education completed……………………………………………………………… 2. How long have you been diagnosed with diabetes and/or hypertension? |

**Section A: Perceived Usefulness and Efficacy of mHealth Intervention**

Question 1: In managing your diabetes and hypertension, how do you perceive the usefulness and importance of having a mobile health app on your phone?

***Probes:***

1a) How has the mobile health app influenced your interactions with healthcare providers?

Can you elaborate on the convenience of using the app for communication with your healthcare providers?

Have you noticed changes in communication dynamics or developed more trust in virtual consultations?

1b) How confident do you feel about sharing information with your provider through the mobile health app?

1c) What training or education did you receive regarding the mHealth app at your facility?

1d) Have you received support from family members in using the app?

**Section B: Quality of mHealth Services and Service Satisfaction**

Question 2: How effectively do you utilize the app's functions, including entering information, responding to reminders, viewing data, and scheduling appointments?

***Probes:***

2a) What specific changes would you consider important for integrating the mobile health app into your routine?

How do you anticipate the app influencing the management of your diabetes and hypertension? What improvements or changes do you see?

2b) How does the app contributes to controlling your blood sugar/blood pressure?

2c) What type of information would you find valuable to access through the mobile health app?

2d) Do you feel more empowered and in control of your health using the mobile health app? Why or why not? Do you feel in control of your health information and have increased self-efficacy in managing your conditions?

2e) Can you share your experiences with remote health monitoring using the app?

Are you satisfied with the remote monitoring features? What benefits have you received from continuous monitoring?

2f) Are there any obstacles or challenges in using the mobile health app?

Could you share any concerns or reservations regarding the app for your care, and how do you think these challenges could be overcome?

**Section C: Factors Influencing the Implementation of mHealth**

Question 3: How easy is the app to use?

Probes:

3a) How easy was it to learn to use the app?

3b) How is the information on the app organized?

3c) What do you like about the app?

3d) How comfortable do you feel using the app?

3e) What is the amount of time involved in using the app?

3f) How do you feel about receiving treatment through the app?

Question 4: Are there any specialized services offered by your hospital through the mobile health app for diabetes and hypertension management?

***Probes:***

4a) How does the availability of these specialized care services through mHealth app affect your in-hospital treatment experience?

How do the specialized care services of mHealth affect the general care of diabetes and hypertension in your facility?

4b) what are some of your reflections on the process and challenges of using mHealth app services?

Where did you face challenges? How was the feedback from the referred center? Was there any coordination with your providers?

Question 5: What recommendations would you suggest for the implementation of a successful mHealth app?

***Probes:***

5a) would you recommend the use of this app to others dealing with diabetes and hypertension?

What improvements would you suggest to enhance the app’s accessibility and usability?
